# The Relationship Between Guilt Feeling and Job Stress in Oncology Nurses: A Cross‐Sectional Study

**DOI:** 10.1002/hsr2.71864

**Published:** 2026-02-23

**Authors:** Sirous Pourkhajoei, Sina Malek Raeesi, Elham Azizpour, Zahra Rohina, Neda Asadi

**Affiliations:** ^1^ Nursing Research Center Kerman University of Medical Sciences Kerman Iran; ^2^ Department of Occupational Health and Safety Engineering, School of Health Isfahan University of Medical Sciences Isfahan Iran; ^3^ Psychiatrist Tehran University of Medical Sciences Tehran Iran; ^4^ Physiology Research Center, Institute of Neuropharmacology Kerman University of Medical Sciences Kerman Iran; ^5^ Reproductive and Family Health Research Center Kerman University of Medical Sciences Kerman Iran

**Keywords:** guilt, job stress, nurse, psychology

## Abstract

**Background and Aims:**

Oncology nurses are frequently exposed to emotionally demanding situations that may compromise their psychological well‐being and the quality of patient care. This study aimed to assess the levels of occupational stress and caregiver guilt among oncology nurses and to explore the relationship between these two variables.

**MethodS:**

This descriptive‐analytical cross‐sectional study was conducted from April to May 2024 among oncology nurses affiliated with the University of Medical Sciences. A total of 140 nurses were selected through convenience sampling. Data collection instruments included the Caregiver Guilt Questionnaire and the Expanded Nursing Stress Scale. Statistical analyses were performed using analysis of variance, independent *t* tests, and multivariate logistic regression to examine associations between stress, guilt, and demographic variables.

**Result:**

The mean score of occupational stress was moderate (2.77 ± 0.56), while caregiver guilt was relatively low (32.63 ± 9.73). A significant positive correlation was found between job stress and guilt (*r* = 0.51, *p* = 0.01). Gender (*p* = 0.001) and level of education (*p* = 0.01) were significantly associated with job stress. Marital status was significantly related to caregiver guilt (*p* = 0.012).

**Conclusion:**

On the basis of the results and considering the significant relationship between job stress and guilt, it is of great importance to employ job stress reduction techniques among oncology nurses. It is necessary for healthcare system policymakers to take action to reduce job stress among nurses by using factors such as eliminating consecutive work shifts, adding welfare services, and improving the physical working environment.

## 1 Introduction

In the last decade, cancer has emerged as one of the major and growing challenges in the field of health. According to global statistics, in 2020, 19.3 million people were diagnosed with cancer, and this number is expected to rise to 28.9 million by 2040 [1]. A cancer diagnosis can lead to a crisis involving both physical and psychological damage in a patient's life. This underscores the critical importance of care provided in oncology departments, where patients often spend the final days of their lives and endure significant pain and suffering [2]. Oncology nurses, as core members of the care team, spend a considerable amount of time with patients [3]. The nursing role for cancer patients is highly stressful [2]. Neglecting this occupational stress can result in serious consequences, including diminished quality of life, emotional exhaustion, and impaired professional performance among nurses [4, 5, 6].

Job stress is a common phenomenon across professions, but it is particularly pronounced in occupations involving intense human interaction [7]. Researchers have identified nursing as one of the most stressful professions [8]. In this context, oncology nurses require special attention. They face numerous emotional challenges, including feelings of helplessness, guilt, and difficulty managing negative emotions [3]. Repeated exposure to patient death and ethical dilemmas can lead to moral distress and intensify feelings of guilt among oncology nurses [7, 9].

Nurses often experience guilt due to a perceived failure to provide adequate care, which can adversely affect their own psychological well‐being [10]. Studies have shown that guilt plays a significant role in negative psychological outcomes; the greater the intensity of guilt, the higher the likelihood of experiencing severe emotional consequences [5, 11].

Moreover, oncology nurses often operate in ethically complex environments where they must balance institutional constraints, patient autonomy, and emotional involvement. These conditions can lead to moral distress, a psychological imbalance that arises when nurses are unable to act according to their ethical beliefs due to external limitations [9]. Moral distress has been linked to increased levels of burnout, emotional fatigue, and even intentions to leave the profession [11]. In high‐intensity settings such as oncology wards, where death and suffering are frequent, the accumulation of unresolved ethical tensions may amplify feelings of guilt and psychological strain. Understanding how job stress interacts with guilt and moral [12]. Distress is essential for developing targeted interventions that support nurse well‐being and improve patient care outcomes [13, 14].

Given the emotional complexity and ethical demands of oncology nursing, there is a pressing need to better understand the psychological mechanisms that affect nurses' well‐being and performance. While previous studies have explored burnout, compassion fatigue, and moral distress in oncology settings [6, 13], few have specifically examined the interplay between job stress and guilt as co‐occurring emotional burdens. Guilt, often overlooked in occupational health research, may act as a mediator or amplifier of stress‐related outcomes, influencing both mental health and care quality [14]. This research seeks to contribute to the growing body of evidence on nurse mental health and to offer insights for policy‐makers and hospital administrators in designing interventions that promote resilience and ethical support in oncology care environments.

### 1.1 Objectives of the Present Study

Determining the average score of job stress and guilt in oncology nurses.

Investigate the relationship between job stress and guilt among oncology nurses.

## 2 Methods

### 2.1 Sample and Setting

This descriptive‐analytical cross‐sectional study was conducted between April and May 2024. Sampling was performed using a convenience method among oncology nurses affiliated with. University of Medical Sciences. The total population of oncology nurses across affiliated hospitals was approximately 230 at the time of data collection. Therefore, the final sample of 140 participants represents approximately 61% of the accessible population.

Inclusion criteria included the absence of any psychological disorder based on self‐report. Additionally, participants were required to have at least 6 months of work experience in oncology wards to ensure adequate exposure to the clinical environment. Exclusion criteria included completing less than two‐thirds of the questionnaire. Participants who completed more than two‐thirds but not the entire questionnaire were included in the analysis, provided that missing data did not affect the scoring of the main variables.

An online survey was conducted through social media platforms (e.g., WhatsApp) and personal contacts. A brief written description of the study and its objectives was sent to all potential participants. On the basis of a pilot study and statistical parameters (correlation coefficient, 95% confidence level, and 90% test power), the required sample size was estimated at 154. Finally, 14 participants were excluded due to incomplete responses, resulting in a final sample size of 140.

### 2.2 Ethics Approval and Consent to Participate

This study was approved by the Ethics Committee of. University of Medical Sciences (Approval No. 404000167 and Ethics Code IR.KMU.REC. 1404.197). All methods were carried out in accordance with relevant guidelines and regulations. Participation in this study was voluntary. All participants were informed about the objectives and procedures of the study, and their written informed consent was obtained.

## 3 Measures

Data were collected using a three‐part questionnaire consisting of demographic information, the Caregiver Guilt Questionnaire (CGQ), and the Expanded Nursing Stress Scale (ENSS).

The demographic section included variables such as age, gender, marital status, education level, and occupational conditions (e.g., shift type, number of night shifts per month, and work experience).

The CGQ, developed by Losada et al. [15], assesses caregiver guilt across five factors using a five‐point Likert scale (0 = never to 4 = always/almost always), with one reverse‐scored item (item 6) and no specific subscales is defined. Total scores range from 0 to 88, with higher scores indicating greater guilt. The CGQ demonstrated good internal consistency, with Cronbach's *α* = 0.88 for the entire scale [15]. To establish reliability, the CGQ was administered to 30 individuals from the target population, yielding Cronbach's *α* = 0.89 for internal consistency.

The ENSS includes 57 items across nine subscales: Death and Dying (seven items), Conflict with Physicians (five items), Inadequate Emotional Preparation (three items), Problems with Peers (six items), Problems with Supervision (seven items), Workload (nine items), Uncertainty Concerning Treatment (nine items), Patients and Their Families (eight items), and Discrimination (three items). Items are rated on a six‐point Likert scale from 0 to 5, where 0 indicates the situation does not apply. Total scores range from 0 to 228, with higher scores indicating greater job stress. Subscale scores are calculated by dividing the total score of each subscale by the number of items. The range of mean values for the total score and subscales is 0–4, and no specific cut‐off point is defined. The ENSS is a nurse‐specific stress assessment instrument that has high validity and reliability, with Cronbach's *α* = 0.96 [16].

### 3.1 Data Analysis

SPSS version 25 was used for statistical analysis. Descriptive statistics were calculated for demographic variables. The normality of data distribution was assessed using the Kolmogorov–Smirnov test (0.05 < *p* value). On the basis of the results, parametric tests were used. Logistic regression analysis was conducted to examine the relationship between job stress and guilt, controlling for relevant demographic and occupational factors. The significance level was set at 0.05.

## 4 Result

The mean age of the study participants was 33.1 years (SD = 6.43). The majority of participants were female (*n* = 125, 89.28%), married (*n* = 109, 77.9%), held a bachelor's degree (*n* = 105, 75%), worked in rotation shifts (*n* = 114, 81.4%), and had between 1 and 5 years of work experience (*n* = 76, 54.28%). Additional demographic and occupational characteristics—including education level, shift type, number of night shifts per month, and years of experience—are detailed in Table 1. These variables were further analyzed in relation to job stress and caregiver guilt scores.

**Table 1:** Participants' characteristics.

Group variable	Frequency (percent)	Job stress	Min–Max	*p* value	Guilt	Min–Max	Statistics test *p* value
*Gender* a							
Woman	125 (89.28)	2.92 ± 0.60	1.80–4.00	< 0.001	38.37 ± 1.81	34–42	0.29
Male	15 (10.71)	2.20 ± 0.36	1.50–2.90		39.60 ± 0.54	38–41	
*Marital status* a							
Married	109 (77.9)	2.14 ± 0.11	1.90–2.40	0.12	39.07 ± 11.45	22–55	0.12
Unmarried	31 (22.1)	2.15 ± 6.86	1.80–2.50		32.81 ± 10.15	22–50	
*Educational level* b							
Bachelor	105 [75]	2.16 ± 0.32	1.80–2.90	0.23	33.57 ± 12.14	22–55	0.89
Master's	30 (21.4)	2.10 ± 0.63	1.70–3.20		34.70 ± 10.37	22–52	
PhD	5 (3.6)	2.97 ± 0.40	2.40–3.50		33.20 ± 7.36	25–45	
*Shift type* a							
Rotation shift	114 (81.4)	2.55 ± 0.89	1.80–4.00	0.54	35.01 ± 11.02	22–55	0.61
Fix shift	26 (18.6)	2.8 ± 0.44	2.10–3.50		28.54 ± 12.75	22–50	
*Number of night shifts* b							
0	20 (14.3)	19.15 ± 5.77	1.80–2.50	0.19	32.40 ± 15.77	22–50	0.31
1–3	8 (5.7)	2.25 ± 0.25	2.00–2.50		33.17 ± 14.78	22–50	
4–6	39 (27.9)	2.94 ± 0.07	2.80–3.10		33.23 ± 10.37	22–50	
7–9	41 (29.3)	2.10 ± 0.10	1.90–2.30		32.95 ± 1.47	30–35	
> 10	32 (22.9)	2.08 ± 0.40	1.80–2.90		34.96 ± 9.75	22–50	
1–5	76 (54.28)	2.32 ± 0.32	1.80–2.90		33.66 ± 11.39	22–55	
*Work experience* a							
5–10	38 (27.14)	2.51 ± 0.70	1.90–3.80	0.17	32.85 ± 11.46	22–55	0.22
> 10	26 (18.57)	2.10 ± 0.53	1.80–3.00		36 ± 11.74	22–55	

^a^ Independent *t* test.

^b^ Analysis of variance.

The mean score of job stress among oncology nurses was 2.77 (SD = 0.56), indicating a moderate level of occupational stress. The mean score of caregiver guilt was 32.63 (SD = 9.73), suggesting a relatively low level of guilt. A statistically significant positive correlation was observed between job stress and guilt (*r* = 0.51, *p* = 0.01), indicating that higher levels of job stress were associated with increased feelings of guilt among participants (Table 2).

**Table 2:** Correlation of job stress subscales and guilt.

	Guilt sore	
Variable	*r*	*p* value
Death and dying	0.61	0.04
Conflict with physicians	0.25	0.005
Inadequate emotional preparation	0.58	0.06
Problems with peers	0.38	0.02
Problems with supervision	0.31	0.007
Workload	0.68	0.03
Uncertainty concerning treatment	0.45	0.0001
Patients and their families	0.49	0.007
Discrimination	0.21	0.001
Total score	0.51	0.01

To further explore the predictors of job stress, a multivariate logistic regression analysis was conducted using the enter method. The results revealed that gender (*β* = – 7.74, *t* = – 2.165, *p* = 0.020) and educational level (*β* = 2.32, *t* = 2.165, *p* = 0.022) were significant predictors of job stress. Specifically, female nurses and those holding a PhD degree reported higher levels of stress. The adjusted *R* ^2^ of the final model was 0.072, indicating that these two variables collectively explained 7.2% of the variance in job stress scores. Full regression coefficients and statistical details are presented in Table 3.

**Table 3:** The logistic model of associations of important variables with job stress.

	Multivariate logistic regression			
Variable	Unstandardized *β*	Standardized coefficients *β*	*t*	*p* value
Age	−0.071	−0.123	−0.730	0.4
Gender	−7.74	−0.247	−2.165	0.02
Marital status	1.42	0.158	1.123	0.25
Educational level	2.32	0.217	2.165	0.022
Shift type	3.54	−0.196	2.197	0.12
Number of night shifts	−0.135	−0.168	−1.751	0.07
Work experience	0.161	0.195	1.09	0.23

Furthermore, the results of the multivariate logistic regression analysis revealed that marital status was a statistically significant predictor of guilt levels among oncology nurses. Specifically, married participants reported higher levels of guilt compared with their unmarried counterparts (*β* = – 6.235, *t* = – 2.367, *p* = 0.010). This suggests that marital responsibilities may intensify emotional burdens in caregiving roles. The adjusted *R* ^2^ of the final model was 0.061, indicating that marital status alone accounted for approximately 6.1% of the variance in guilt scores. Although this effect size is modest, it highlights the relevance of personal life context in shaping psychological responses to occupational stress. Detailed regression coefficients for all variables are presented in Table 4.

**Table 4:** The logistic model of associations of important variables with guilt.

	Multivariate logistic regression			
Variable	Unstandardized *β*	Standardized coefficients *β*	*t*	*p* value
Age	−0.232	−0.129	−0.879	0.2
Gender	3.726	0.073	0.479	0.71
Marital status	−6.235	−0.218	−2.367	0.01
Educational level	0.854	0.063	0.342	0.56
Shift type	3.725	0.167	1.321	0.16
Number of night shifts	−0.189	−0.093	−1.017	0.21
Work experience	0.037	0.018	0.140	0.89

## 5 Discussion

This study investigated the psychological dimensions of job stress and feelings of guilt among oncology nurses. The findings revealed that participants experienced a moderate level of job stress and a relatively low level of guilt. Notably, the mean scores for both constructs were slightly higher than those reported in a similar study conducted in Portugal [17], suggesting contextual or cultural factors may influence emotional responses in oncology settings.

The result shows that guilt tends to increase in tandem with rising job stress levels. This relationship supports previous research showing that oncology nurses often internalize emotional burdens due to their exposure to patient suffering, ethical dilemmas, and end‐of‐life care [7]. Maunder et al. [18] stated that such emotional strain can lead to moral distress, where nurses feel unable to meet their own ethical standards, resulting in guilt and psychological fatigue. Unresolved emotional responses have been linked to burnout, characterized by emotional exhaustion and reduced personal accomplishment [18]. Moreover, job stress has been associated with diminished caring behaviors, which may further reinforce feelings of guilt and inadequacy [1].

These findings underscore the importance of addressing stress and guilt as interconnected phenomena that can undermine nurse well‐being and compromise the quality of patient care. To mitigate these effects, healthcare institutions should implement targeted interventions such as resilience training, structured emotional debriefings, and access to psychological support services [19]. Recent studies have shown that resilience‐building programs can reduce turnover intention and improve emotional regulation among oncology nurses [20].

Gender was found to be a significant predictor of job stress, with female nurses reporting higher stress levels than males. This may be attributed to the dual burden of professional and domestic responsibilities, as well as gendered coping strategies. Women are more likely to employ emotion‐focused coping mechanisms, which may intensify psychological distress in high‐pressure environments [21]. These findings align with studies showing that female nurses experience higher levels of stress related to interpersonal relationships and emotional labor [22, 23]. In contrast, men tend to use problem‐focused strategies and may externalize stress through physical symptoms. Although these gender differences are well‐documented, some studies have reported no significant differences, possibly due to sample composition or cultural norms.

Educational level also emerged as a significant predictor of job stress, with nurses holding PhD degrees reporting higher stress levels. This contrasts with findings by Magakwe et al., who found that higher educational attainment emerged as a significant protective factor against depression and anxiety, with postgraduate qualifications associated with lower odds compared with a Bachelor's degree [22]. One possible explanation is that higher educational attainment may be linked to increased responsibilities, expectations, or role ambiguity in clinical settings. Continuing education and familiarity with technological equipment have been proposed as strategies to reduce stress levels [24]. These findings suggest that advanced qualifications may not always buffer stress, and tailored support is needed for nurses in leadership or academic roles.

In this study, married nurses reported higher levels of guilt. This may reflect the challenge of balancing professional duties with family obligations, leading to role conflict and emotional strain. Previous studies have shown that marital satisfaction can be negatively affected by shift work and occupational demands [21]. The dual expectations of caregiving at work and at home may place married nurses at greater risk of emotional overload, especially when institutional support is lacking. This highlights the need for family‐friendly workplace policies and flexible scheduling to reduce emotional strain and improve work‐life balance.

No significant associations were found between age and either stress or guilt, which is consistent with several prior studies [25, 26]. Although age is often considered a factor in emotional resilience, the lack of association in this study may reflect the homogenizing effect of professional demands in oncology settings. Similarly, shift type did not significantly influence stress or guilt levels. While some literature suggests that night shifts are associated with increased stress and health risks [27, 28]. These discrepancies may be due to differences in institutional support, staffing patterns, or cultural norms regarding shift work. Future studies should consider stratifying shift types more precisely (e.g., rotating vs. permanent night shifts) to better capture their psychological impact.

Work experience was not significantly related to stress or guilt in this study, although previous research has linked longer tenure to higher burnout rates. Babapour et al. [21] found that with increasing work experience, the rate of burnout was higher, and there was a relationship between job stress and job burnout. Long‐term job stress has been shown to lead to burnout, which is often used interchangeably with occupational stress among healthcare personnel [29, 30]. Some studies suggest that while novice nurses may experience acute stress due to a lack of confidence, experienced nurses may face chronic stress from cumulative exposure to emotionally demanding situations [31, 32]. This highlights the importance of tailored support strategies across career stages.

Theoretically, this study contributes to models of moral distress and emotional labor in nursing by demonstrating how job stress and guilt interact in oncology settings. It supports the notion that psychological burdens in caregiving professions are multidimensional and often interrelated. From a practical perspective, results can inform targeted stress management interventions or training programs for caregivers, especially focusing on gender and education differences. Health organizations or HR departments could design support systems and counseling services to reduce both occupational stress and associated guilt among caregivers. Marital status findings might guide family‐based mental health interventions, encouraging spouse involvement in caregiver support.

Despite these valuable insights, the study has several limitations. First, the use of convenience sampling and self‐report measures may limit generalizability and introduce response bias. Second, the cross‐sectional design precludes causal inference. Third, the study did not account for organizational variables such as staffing ratios, managerial support, and departmental workload, which may significantly influence stress and guilt levels.

Future research should employ longitudinal designs to examine changes over time and explore the effectiveness of targeted interventions. Qualitative studies may also provide deeper insight into the lived experiences of oncology nurses and the contextual factors shaping their emotional responses. Comparative studies across different hospital departments and cultural settings could help identify universal versus context‐specific stressors. Additionally, integrating organizational metrics and psychological screening tools may enhance the precision and applicability of future findings.

## 6 Conclusion and Recommendations

This study highlights the complex interaction between job stress and guilt among oncology nurses, a group that faces unique emotional and ethical challenges in their daily practice. The significant correlation found between job stress and guilt suggests that psychological strain in oncology settings goes beyond physical workload and encompasses the ethical and emotional dimensions of caregiving. Results suggest that individual and professional factors—such as gender, education level, and marital status—can play an important role in shaping the psychological experiences of oncology nurses. Consequently, implementing targeted psychosocial interventions, emotional support programs, and educational initiatives tailored to the specific needs of this group could help reduce negative emotional outcomes among oncology nursing staff. It also leads to improved quality of patient care and organizational efficiency.

The original contribution of this study is to provide new evidence for specific demographic factors that determine the psychological vulnerability of oncology nurses. These insights can inform future policy and intervention design in oncology nursing.

## Author Contributions

Neda Asadi, Sirous Pourkhajoei, and Zahra Rohina conceptualized and designed the study, supervised all phases of the research, including formative assessment, instrument development, data analysis, and interpretation. They also contributed to drafting and revising the manuscript. Sina Malek Raeesi and Elham Azizpour were responsible for data collection, preliminary analysis, and preparation of the initial manuscript draft. All authors reviewed and approved the final version of the article.

## Funding

The authors received no specific funding for this work.

## Ethics Statement

This study was approved by the Ethics Committee of the University of Medical Sciences with (Approval No. 404000167 and Ethics Code IR.KMU.REC. 1404.197). All methods were carried out in accordance with relevant guidelines and regulations. Participation in this study was voluntary. All participants were informed about the objectives and the study process, and their informed consent was obtained.

## Consent

The authors have nothing to report.

## Conflicts of Interest

The authors declare no conflicts of interest.

## Transparency Statement

The lead author Neda Asadi affirms that this manuscript is an honest, accurate, and transparent account of the study being reported; that no important aspects of the study have been omitted; and that any discrepancies from the study as planned (and, if relevant, registered) have been explained.

## Data Availability

The data are available upon request from the corresponding author after signing appropriate documents in line with the ethical application and the Ethics Committee's decision.
