# Correction to “The Relationship Between Guilt Feeling and Job Stress in Oncology Nurses: A Cross‐Sectional Study”

**DOI:** 10.1002/hsr2.72753

**Published:** 2026-07-01

Pourkhajoei S, Malek Raisi S, Azizpour E, Rohina Z, Asadi N. The Relationship Between Guilt Feeling and Job Stress in Oncology Nurses: A Cross‐Sectional Study. Health Sci Rep. 2026;9(2):e71864. https://doi.org/10.1002/hsr2.71864

The name of the second author was incorrect and has been updated from Sina Malek Raeesi to Sina Malek Raisi in the author list and the Author Contributions section.

The online version of this article has been corrected accordingly.

We apologize for this error.

